# Laparoscopic management of acute appendicitis in situs inversus

**DOI:** 10.4103/0972-9941.28184

**Published:** 2006-12

**Authors:** Vishwanath Golash

**Affiliations:** Department of Surgery, Sultan Qaboos Hospital, Salalah - 211, Sultanate of Oman

**Keywords:** Laparoscopy, situs inversus, appendicitis

## Abstract

Situs inversus is often detected incidentally in adults during imaging for a acute surgical emergency. We present a case of acute appendicitis in an adult who was previously unaware about his situs anomaly. A laparoscopic approach is helpful to deal with this condition. A 40 year old man was admitted with history of acute left lower abdominal pain, with uncontrolled diabetic keto-acidosis. Clinically, he was diagnosed as acute diverticulitis with localized peritonitis. Subsequent imaging studies and laparoscopy confirmed the diagnosis of situs inversus and acute left- sided appendicitis.

He successfully underwent laparoscopic appendectomy. His postoperative recovery was uneventful. Although technically more challenging because of the reverse laparoscopic view of the anatomy, the laparoscopic diagnosis and management of acute appendicitis is indicated in situs inversus.

## INTRODUCTION

The situs anomalies are rare congenital defects and may go unrecognized until incidentally detected during imaging for unrelated conditions or during emergency surgery. Laparoscopy is indicated in these patients, as the clinical and imaging findings can be confusing in conjunction with acquired diseases. There have been only two previous case reports of laparoscopic appendectomy in situs inversus.[1]

## CASE REPORT

**Figure 1:**
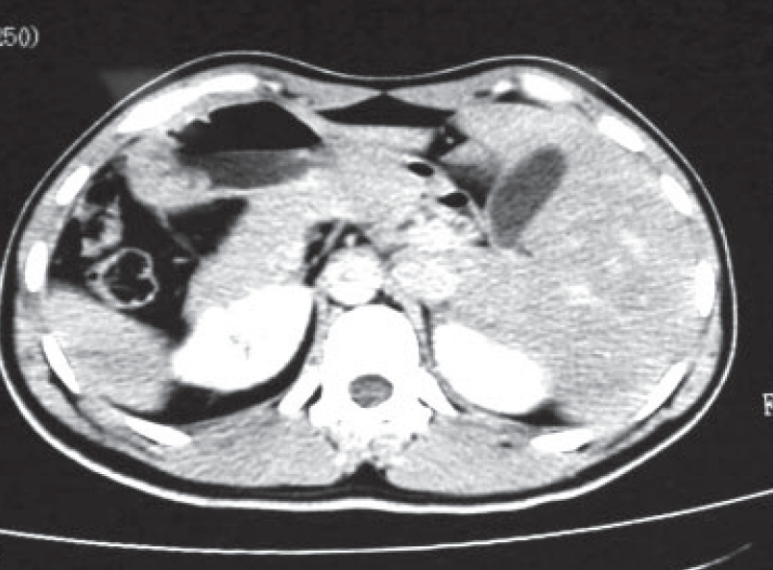
CT scan of situs inversus

A 40 year old man was admitted with history of left lower abdominal pain, fever and vomiting for the past two days. On examination, he was febrile with a temperature of 38.3°C and moderately dehydrated. There was tenderness with rebound tenderness in the left iliac fossa. Clinically he was diagnosed as acute diverticulitis. His subsequent blood investigations data were as follows: Urea 5.67 mmol/L (range 2.1-7.1), Creatinine 96.6 mmol/L (range 62-106), Glucose 22 mmol/L (range 3.6-5.5), Sodium 135, Potassium 5.1 and white cell count 16.1 10^3/µL (range 4.0-11.0). The blood gases analysis was suggestive of metabolic acidosis with a pH of 7.2 and decrease in PCO2. The urine examination showed three plus of ketone. A routine ultrasound examination of abdomen was requested, which revealed the liver on the left side, spleen on the right side and an inflammatory mass in the left iliac fossa. This changed the diagnosis to situs inversus and the inflammatory mass in the left iliac fossa was now thought to be appendicular in origin. The heart sounds were heard over the right chest and subsequent plain X-ray of chest showed dextrocardia. The ECG findings were suggestive of dextrocardia, sinus rhythm and intra-ventricular conduction defect. The CT with contrast confirmed the diagnosis of complete situs inversus with dextrocardia and the appendix was seen as a tubular structure in the left iliac fossa, in front of left psoas muscle with surrounding inflammation [Figure 1]. The patient was unaware of his diabetes mellitus and his situs inversus anomaly. He was managed with intravenous fluids, insulin infusion and parental antibiotics prior to his surgery.

**Figure 2:**
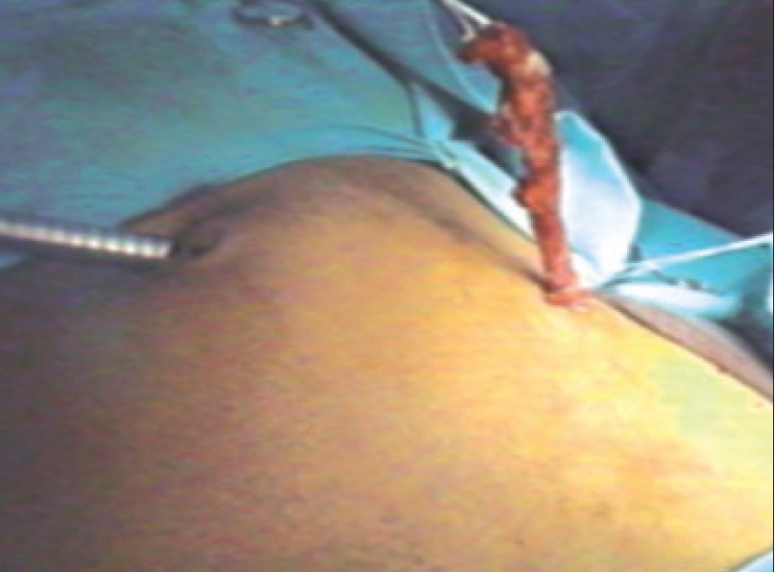
Extracorporeal appendetomy in left iliac fossa

At laparoscopy, the situs inversus findings were confirmed. The cecum and ascending colon was on the left side. An inflammatory mass was seen in left iliac fossa, covered with omentum. Another 10 mm port was inserted in the left iliac fossa and a 5 mm port in the suprapubic region. The omentum was separated from the mass; the appendix was laying partially retrocecal and acutely inflamed, but not perforated. The appendicular pedicle was ligated intracorporeally. The mesentery of the appendix was thick and bulky; the intracorporeal ligation of the appendicular pedicle helped in mobilizing the appendix and its delivery through the left iliac port. The appendectomy was performed extracorporeally [Figure 2].

## DISCUSSION

The incidence of situs anomalies reported in the literature, varies from 0.001 to 0.01% in the general population. The overlapping features of some situs anomalies and the presence of acute acquired diseases may result in confusing imaging findings. Diagnosis of acute disease processes is challenging in these patients due to altered anatomy.[2] The differential diagnosis in situs inversus patients may not be readily seen in the emergency settings and is often delayed as a result of lack of uniformity in physical signs.[3,4] Although the viscera are transposed, it is thought that the central nervous system may not share the reverse transposition, leading to confusing symptoms and signs. The pain of the left-sided appendicitis has been reported to the right iliac fossa in about 50% of the patients of situs inversus. The pain and tenderness in the left iliac fossa can also be due to a right-sided, long, dilated appendix located in the left lower quadrant. Laparoscopy helps in identifying and treating acute surgical emergencies quickly and efficiently, when the clinical and imaging studies are difficult to interpret in situs anomalies.

It is our routine practice to remove the appendix in all cases of diagnostic laparoscopy (so as to avoid future diagnostic confusions). Wherever appendectomy was performed, it was accomplished via an assisted two-port method in most cases.[5]
